# Supplementary material for: Genome-Wide Identification of Novel miRNAs and Infection-Related Proteins in Leishmania major via Comparative Analysis of the Protozoa, Vectors, and Mammalian Hosts
Source: Pathogens. 2025 Oct 21;14(10):1068. doi: 10.3390/pathogens14101068 (PMC12567135; doi:10.3390/pathogens14101068)
Supplement: Supplementary file 1 [file pathogens-14-01068-s001.zip › pathogens-3912889-supplementary.pdf]

No.	Accession number	Current annotation	Annotated protein	Gene symbol	E-value
1	XP_001686672.1	conserved hypothetical protein	Intraflagellar transport protein 122 homolog	IFT122	2.27E-149
2	XP_888594.1	conserved hypothetical protein	TTC21B protein (Tetratricopeptide repeat domain 21B)	TTC21B	1.57E-117
3	XP_001684649.1	conserved hypothetical protein	Bardet-Biedl syndrome 2 protein homolog	Bbs2	2.43E-103
4	XP_001686229.1	conserved hypothetical protein	Mitochondrial chaperone BCS1 (h-BCS1) (BCS1-like pr	BCS1L	1.14E-83
5	XP_001685262.1	conserved hypothetical protein	Endoplasmic reticulum-Golgi intermediate compartment	ERGIC3	1.49E-77
6	XP_001686195.1	conserved hypothetical protein	Carnosine N-methyltransferase (EC 2.1.1.22)	CARNMT1	7.04E-72
7	XP_001682610.1	conserved hypothetical protein	Intraflagellar transport 80	IFT80	2.75E-68
8	XP_003722084.1	conserved hypothetical protein	WD repeat domain 34	DYNC2I2	2.63E-66
9	XP_003722065.1	conserved hypothetical protein	Cilia and flagella associated protein 58	CFAP58	5.87E-61
10	XP_001682381.1	conserved hypothetical protein	Tetratricopeptide repeat protein 27	TTC27	1.01E-58
11	XP_001684329.1	conserved hypothetical protein	Zinc finger MYND domain-containing protein 10	ZMYND10	3.96E-56
12	XP_001680824.1	conserved hypothetical protein	Basic immunoglobulin-like variable motif-containing pro	BIVM	5.13E-56
13	XP_001683666.1	conserved hypothetical protein	PWP1 homolog, endonuclein	PWP1	8.23E-54
14	XP_003722868.1	conserved hypothetical protein	Hypoxia up-regulated protein 1	HYOU1	9.27E-53
15	XP_001682917.1	conserved hypothetical protein	tRNA (uracil(54)-C(5))-methyltransferase (EC 2.1.1.35)	TRMT2B	6.68E-52
16	XP_003722829.1	conserved hypothetical protein	Haloacid dehalogenase-like hydrolase domain-containing	HDHD5	3.33E-50
17	XP_001686507.1	conserved hypothetical protein	Ufm1-specific protease 2	UFSP2	1.20E-46
18	XP_001681666.1	conserved hypothetical protein	Cell division cycle protein 23 homolog (Anaphase-prom	CDC23	2.55E-46
19	XP_001681891.1	conserved hypothetical protein	HECT domain E3 ubiquitin protein ligase 3	HECTD3	3.73E-39
20	XP_001681297.1	conserved hypothetical protein	Senataxin	SETX	2.43E-38
21	XP_001683338.1	conserved hypothetical protein	Leucine-rich repeat-containing protein 34	Lrrc34	3.49E-36
22	XP_001682417.1	conserved hypothetical protein	Transmembrane and coiled-coil domains 4	TMCO4	4.93E-35
23	XP_003721582.1	conserved hypothetical protein	TBC1 domain containing kinase	TBCK	2.69E-33
24	XP_001687518.1	conserved hypothetical protein	Cell division cycle protein 27 homolog	CDC27	2.96E-31
25	XP_001687012.1	conserved hypothetical protein	Crooked neck pre-mRNA splicing factor 1	CRNKL1	9.04E-31
26	XP_001684907.1	conserved hypothetical protein	UDP-N-acetylglucosamine--peptide N-acetylglucosamin	OGT	2.01E-28
27	XP_001682966.1	conserved hypothetical protein	Vacuolar protein sorting-associated protein 51 homolog	VPS51	6.27E-28
28	XP_001682555.1	conserved hypothetical protein	Dynein 2 intermediate chain 1	DYNC2I1	1.07E-26
29	XP_001685647.1	conserved hypothetical protein	Intraflagellar transport 22	IFT22	7.28E-26
30	XP_003721983.1	conserved hypothetical protein	Cell wall biosis 43 C-terminal homolog	CWH43	1.41E-25
31	XP_001684935.1	conserved hypothetical protein	Ankyrin 2	ANK2	1.02E-24
32	XP_001686460.1	conserved hypothetical protein	Ankyrin-1 (ANK-1) (Ankyrin-R) (Erythrocyte ankyrin)	ANK1	2.52E-24
33	XP_001687034.1	conserved hypothetical protein	Cilia and flagella associated protein 298	CFAP298	3.66E-24
34	XP_001683473.1	conserved hypothetical protein	Exonuclease 1 (EC 3.1.-.-)	Exo1	2.08E-23
35	XP_003722883.1	conserved hypothetical protein	Small glutamine rich tetratricopeptide repeat containing a	SGTA	5.00E-20
36	XP_001683727.1	conserved hypothetical protein	Leucine zipper like transcription regulator 1	LZTR1	6.03E-20
37	XP_001682530.1	conserved hypothetical protein	Protein kintoun (Dynein assembly factor 2, axonemal)	DNAAF2	2.47E-19
38	XP_001684126.1	conserved hypothetical protein	Dynein axonemal assembly factor 1	Dnaaf1	3.35E-19
39	XP_001684820.1	conserved hypothetical protein	WD repeat domain 27	WDR27	2.82E-18
40	XP_001681455.1	conserved hypothetical protein	Exportin 7 (XPO7 protein)	XPO7	1.37E-17
41	XP_001682380.1	conserved hypothetical protein	Transmembrane protein 94	TMEM94	3.87E-17
42	XP_003722189.1	conserved hypothetical protein	Ankyrin-3 (ANK-3) (Ankyrin-G)	ANK3	5.84E-17
43	XP_001685977.1	conserved hypothetical protein	Magnesium-dependent phosphatase 1 (MDP-1) (EC 3.1.3	MDP1	9.22E-17
44	XP_001684589.1	conserved hypothetical protein	Mucin-1 (MUC-1) (Breast carcinoma-associated antigen	MUC1	3.67E-16
45	XP_001685260.1	conserved hypothetical protein	RCC1 and BTB domain containing protein 2	RCBTB2	2.05E-15
46	XP_001682168.1	conserved hypothetical protein	Ribosomal RNA processing 1	RRP1	2.86E-15
47	XP_001684346.1	conserved hypothetical protein	RING-type E3 ubiquitin transferase (EC 2.3.2.27)	MIB2	3.65E-15
48	XP_003722004.1	conserved hypothetical protein	Adenylate kinase 7	AK7	5.41E-15
49	XP_001684669.1	conserved hypothetical protein	ARMC6 protein (Armadillo repeat containing 6)	ARMC6	4.83E-14
50	XP_001683655.1	conserved hypothetical protein	Centrin-1	CETN1	3.37E-13
51	XP_001685005.1	conserved hypothetical protein	Polyribonucleotide 5'-hydroxyl-kinase Clp1 (EC 2.7.1.78	CLP1	8.20E-13
52	XP_003722527.1	conserved hypothetical protein	Iron-sulfur cluster assembly 2	ISCA2	1.81E-12
53	XP_003722169.1	conserved hypothetical protein	tRNA dimethylallyltransferase (EC 2.5.1.75) (Isopenteny	Trit1	2.70E-12
54	XP_001682516.1	conserved hypothetical protein	Ankyrin repeat domain 6	ANKRD6	8.90E-12
55	XP_001682571.1	conserved hypothetical protein	Golgi transport 1B	GOLT1B	1.34E-11
56	XP_001682348.1	conserved hypothetical protein	NLR family, CARD domain containing 3	NLRC3	1.74E-11
57	XP_001683470.1	conserved hypothetical protein	RPTOR independent companion of MTOR complex 2	RICTOR	3.42E-11

58	XP_888619.1	conserved hypothetical protein	Transcription factor 25	TCF25	4.85E-11
59	XP_001682271.1	conserved hypothetical protein	Centrosomal protein of 120 kDa (Cep120) (Coiled-coil d	Cep120	4.89E-11
60	XP_003722462.1	conserved hypothetical protein	UTP20 small subunit processome component	UTP20	6.49E-11
61	XP_001682860.1	conserved hypothetical protein	GATOR2 complex protein WDR24	WDR24	7.93E-11
62	XP_001682467.1	conserved hypothetical protein	FYVE, RhoGEF and PH domain containing 2	Fgd2	1.31E-10
63	XP_001682088.1	conserved hypothetical protein	MORN repeat-containing protein 4 (Retinophilin)	MORN4	2.17E-10
64	XP_003721664.1	conserved hypothetical protein	Sperm associated antigen 1	SPAG1	3.42E-10
65	XP_001685415.2	conserved hypothetical protein	Abelson helper integration site 1	AHI1	4.77E-10
66	XP_001687072.1	conserved hypothetical protein	Sugar transporter SWEET	SLC50A1	5.17E-10
67	XP_001685816.1	conserved hypothetical protein	WD repeat-containing protein 70	WDR70	9.96E-10
68	XP_001687680.1	conserved hypothetical protein	Pericentrin/AKAP-450 centrosomal targeting domain-co	PCNT	1.51E-09
69	XP_001686369.1	conserved hypothetical protein	Vacuolar protein sorting-associated protein 52 homolog	VPS52	2.15E-09
70	XP_001681996.1	conserved hypothetical protein	Rab proteins geranylgeranyltransferase component A	CHM	2.65E-09
71	XP_003722521.1	conserved hypothetical protein	Alkylated DNA repair protein alkB homolog 8 (EC 2.1.1	ALKBH8	2.82E-09
72	XP_001686934.1	conserved hypothetical protein	Translocase of outer mitochondrial membrane 34	TOMM34	4.21E-09
73	XP_001682176.1	conserved hypothetical protein	Laminin subunit alpha-5 (Laminin-10 subunit alpha) (La	LAMA5	5.02E-09
74	XP_001683063.1	conserved hypothetical protein	Abnormal spindle-like microcephaly-associated protein h	Aspm	6.46E-09
75	XP_001681849.1	conserved hypothetical protein	NME/NM23 family member 5	NME5	1.43E-08
76	XP_001683970.1	conserved hypothetical protein	Stress-induced-phosphoprotein 1 (Hsc70/Hsp90-organizi	STIP1	1.52E-08
77	XP_001684817.1	conserved hypothetical protein	Mitochondrial assembly of ribosomal large subunit 1	MALSU1	1.90E-08
78	XP_001685046.1	conserved hypothetical protein	SECIS binding protein 2 like	SECISBP2L	5.55E-08
79	XP_003722642.1	conserved hypothetical protein	General vesicular transport factor p115	USO1	5.65E-08
80	XP_001682120.1	conserved hypothetical protein	Glutamate decarboxylase 2 (EC 4.1.1.15) (65 kDa glutan	GAD2	7.78E-08
81	XP_001685074.1	conserved hypothetical protein	CFAP97 domain containing 2	CFAP97D2	1.05E-07
82	XP_001681003.1	conserved hypothetical protein	DEP domain containing 5, GATOR1 subcomplex subuni	DEPDC5	1.18E-07
83	XP_001683258.1	conserved hypothetical protein	Formin 2	FMN2	1.26E-07
84	XP_003722428.1	conserved hypothetical protein	Ankyrin repeat and sterile alpha motif domain containing	ANKS4B	1.53E-07
85	XP_001686209.1	conserved hypothetical protein	Homer scaffold protein 2	C5H15orf40	1.60E-07
86	XP_001683861.1	conserved hypothetical protein	non-specific serine/threonine protein kinase (EC 2.7.11.1	MAST4	1.68E-07
87	XP_001685795.1	conserved hypothetical protein	Nuclear transcription factor, X-box binding-like 1	Nfxl1	1.77E-07
88	XP_001687546.1	conserved hypothetical protein	Sperm tail PG-rich repeat containing 2	STPG2	3.84E-07
89	XP_001684263.1	conserved hypothetical protein	Histone-lysine N-methyltransferase (EC 2.1.1.364)	KMT2A	4.98E-07
90	XP_001685434.1	conserved hypothetical protein	Protein phosphatase 1 regulatory subunit 16B	PPP1R16B	5.25E-07
91	XP_001681416.1	conserved hypothetical protein	Tetratricopeptide repeat domain 6	TTC6	6.03E-07
92	XP_001685813.1	conserved hypothetical protein	Serine/threonine-protein phosphatase (EC 3.1.3.16)	PPP5C	7.43E-07
93	XP_001682830.1	conserved hypothetical protein	Protein misato homolog 1	MSTO1	8.07E-07
94	XP_001680881.1	conserved hypothetical protein	Progesterone immunomodulatory binding factor 1	PIBF1	8.88E-07
